# Impact of Epithelial Cell Shedding on Intestinal Homeostasis

**DOI:** 10.3390/ijms23084160

**Published:** 2022-04-09

**Authors:** Phuong A. Ngo, Markus F. Neurath, Rocío López-Posadas

**Affiliations:** 1 Department of Medicine 1, Friedrich-Alexander-Universität Erlangen-Nürnberg, 91054 Erlangen, Germany; phuong.ngoanh@uk-erlangen.de (P.A.N.); markus.neurath@uk-erlangen.de (M.F.N.); 2 Deutsches Zentrum Immuntherapie (DZI), 91054 Erlangen, Germany

**Keywords:** epithelial cell shedding, cell extrusion, leaky gut, inflammatory bowel disease (IBD), colorectal cancer

## Abstract

The gut barrier acts as a first line of defense in the body, and plays a vital role in nutrition and immunoregulation. A layer of epithelial cells bound together via intercellular junction proteins maintains intestinal barrier integrity. Based on a tight equilibrium between cell extrusion and cell restitution, the renewal of the epithelium (epithelial turnover) permits the preservation of cell numbers. As the last step within the epithelial turnover, cell shedding occurs due to the pressure of cell division and migration from the base of the crypt. During this process, redistribution of tight junction proteins enables the sealing of the epithelial gap left by the extruded cell, and thereby maintains barrier function. Disturbance in cell shedding can create transient gaps (leaky gut) or cell accumulation in the epithelial layer. In fact, numerous studies have described the association between dysregulated cell shedding and infection, inflammation, and cancer; thus epithelial cell extrusion is considered a key defense mechanism. In the gastrointestinal tract, altered cell shedding has been observed in mouse models of intestinal inflammation and appears as a potential cause of barrier loss in human inflammatory bowel disease (IBD). Despite the relevance of this process, there are many unanswered questions regarding cell shedding. The investigation of those mechanisms controlling cell extrusion in the gut will definitely contribute to our understanding of intestinal homeostasis. In this review, we summarized the current knowledge about intestinal cell shedding under both physiological and pathological circumstances.

## 1. Introduction

### Epithelial Turnover and Cell Shedding

It has been known for more than half a century that intestinal epithelial lining is continuously replaced via the epithelial turnover process [1], whereby cell formation is equal to cell loss. Every few days, Intestinal Epithelial Cells (IECs) are eliminated to exclude aged and potentially damaged cells, in order to warrant the impermeable barrier. In fact, the rapid renewal of the epithelium can be understood as a defense mechanism in response to constant interactions with harmful luminal contents [2]. Joseph Paneth was the first scientist proposing that cells at the crypt and villus are derived from the same embryological origin [3]. After that, the flow of cell movement from crypts to villi has been confirmed in several studies, corroborating that stem cells reside permanently at the base of crypt [4]. Until now, it is clear that in the small intestine crypts comprise stem cells and Paneth cells since those cells do not migrate upwards, while the villi contain enterocytes (covering most part of the epithelium) and other differentiated cells (enteroendocrine, goblet, tuft cells, M cells). Globally, the turnover process comprises four phases: cell proliferation, migration, differentiation, and extrusion/death. Starting with cell proliferation/division at the stem cell compartment (crypt base), the daughter cells then migrate in the “Transit-Amplifying” zone through epithelial–substratum interactions between integrins, heparin sulfate proteoglycans, and the extracellular matrix [5]. Even though this migration was believed to be a consequence of villi contraction (through villous smooth muscle) [6], a recent study suggested that passive mitotic pressure from cell proliferation is the primary force driving cell migration [7]. As they continue to move towards the villus tips, migrating cells also differentiate into various cell subtypes serving different functions (absorption and secretion). At the end of the process, cells are shed out into the lumen with the help of tight-junctional protein rearrangement of neighboring cells, the so-called zipper effect (epithelial cell shedding or apical cell extrusion) [8]. Upon extracellular matrix (ECM) detachment, cells undergo an apoptotic process called anoikis, which is essential in order to avoid shed cells colonizing elsewhere. 

**Figure 1:**
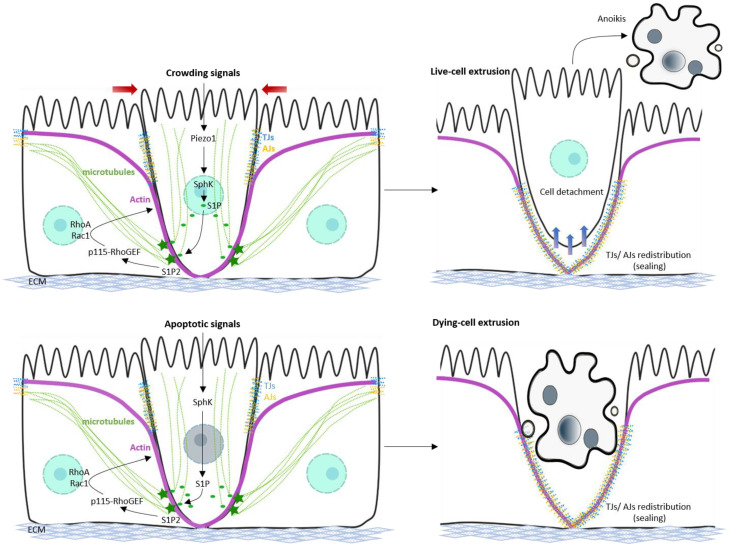
Live versus apoptotic cell shedding.

Cell shedding is considered a key process for tissue turnover in several organs in order to ensure tissue integrity and homeostasis. Cell shedding was first referred to as the “extrusion zone” at Leblond’s lab when they detected irregular cell loss at the villus tips in mouse gut upon interrupted mitosis [9]. Cell extrusion is a multicellular process, which requires dynamic interaction of extruded and surrounding cells to allow cell detachment and release from the ECM. Indeed, the contribution of the shed versus the neighbouring cells in the final outcome is still controversial. Whether non-apoptotic or apoptotic cells are extruded into the lumen defines two types of epithelial cell shedding (Figure 1). Non-apoptotic shedding occurs at areas with the most cell packing, i.e., at the villus tips, and hence is also referred to as crowding-induced cell extrusion or physiological cell shedding. It consists of the extrusion of living cells, which is triggered by stretch-activated channels (Piezo1) due to mechanical cues, followed by the release of Sphingolipid 1 phosphate (S1P), Rho pathway activation, and actin/myosin contractile force [10,11,12]. Upon extrusion, the shed cell undergoes anoikis due to the loss of focal adhesions and the lack of survival signals. On the other hand, apoptotic or cell death-induced cell extrusion is induced by external factors, such as TNF binding to corresponding receptors [13]. In this case, cell apoptosis is well-known to be associated with cell shedding, but whether it initiates the process or occurs as a result of the loss of survival signal upon detachment is unclear [14]. Upon induction of apoptosis, the dying cell contracts and releases molecular signals to activate the formation of actin/myosin II ring in neighboring cells, which squeezes the dying cell out, following a similar mechanism as in non-apoptotic shedding [15]. The “zipper model” suggested that cells about to be shed undergo redistribution of tight Junctions (TJs) and integrins, in order to allow detachment from the ECM and neighboring cells [16]. In addition, dynamic cytoskeletal rearrangement and TJs redistribution of neighboring cells permits the quick sealing of the space vacated by the shed cell, and is therefore important in order to avoid any transient gap in the epithelial sheet [17,18]. Different mechanisms involved in cell shedding are described in detail from chapter III to VI (Figure 1). Despite the physiological relevance of this process, several aspects of cell extrusion remain enigmatic, especially in in vivo situation and in mammals.

## 2. Pathological Relevance of Cell Shedding

Cell extrusion regulates epithelial cell numbers, integrity, and barrier function. Thus, alterations of epithelial cell extrusion leading to the invasion of pathogens have been associated to several pathologies. Besides this, epithelial cell shedding acts as a defense mechanism to remove “damaged” cells whose altered function might lead to pathological consequences, such as infected cells [19] or cells carrying mutations. The so-called “Epithelial Defense against Cancer, EDAC” represents a key mechanism for avoiding cancer [20]. Thus, epithelial cell shedding emerged as a key player in the context of inflammation, infection, and cancer.

### 2.1. Infection

IEC extrusion occurs millions of times per day [21], making the villus tip an attracting zone for a variety of bacteria, virus, and macromolecules to penetrate into the organism. Taking into account that the gut lumen is populated by approximately 38 trillion bacterial cells [22], cell shedding must be tightly regulated in a way that prevent pathogen invasion. Understanding how pathogens detect penetrable sites and break the epithelial layer to access the body might reveal key information behind cell extrusion and related mechanisms.

Many microbes entry the body by exploiting the apical-junctional complex of IECs [23]. In order to access the basolateral domain, some bacteria have developed their own mechanisms to overcome lateral TJs–for example, Vibrio cholerae [24], Helicobacter pylori [25], and *E. coli* [26]–while others highjack epithelial cell extrusion to perpetuate the invasion. The Gram-positive bacterium *Listeria monocytogenes* is an example of a pathogen that takes advantage of cell extrusion to invade the host cell, instead of breaking or modifying TJs. *L. monocytogenes* is a source of fatal opportunistic infection, mostly affecting people with weakened immune system such as pregnant women, elderly people, neonates, and immunocompromised patients. *L. monocytogenes* transmits from contaminated food and spreads to the liver, spleen, central nervous system, and placenta, causing a range of severe clinical manifestations including abortion, sepsis, and meningoencephalitis [27]. *L. monocytogenes* was found to invade MDCK epithelial monolayer at sites where cell extrusion happened, determined by the presence of cells missing nuclei and with the typical funnel-shaped of ZO-1 (shedding cells) (see chapter VIb) [28]. During cell extrusion, E-cadherin is transiently exposed to the apical surface, which enables bacterial InIA and InIB to bind to the N-terminal extracellular domain of E-cadherin and c-Met, respectively [28]. This strategy allows *L. monocytogenes* to colonize the host cells. Interestingly, the authors observed a similar phenomenon in rabbit ideal loops, where *L. monocytogenes* entry occurred at the junctions surrounding extruding cells, mostly around the villus tips [28]. 

*Shigella flexneri* infection affects the terminal ileum, colon, and rectum, causing watery diarrhea followed by dysentery. This disease is particularly dangerous because it occurs in outbreaks with a high mortality rate, and importantly, no licensed vaccine is available [29]. This bacterium has developed another mechanism to make host cells more vulnerable to its invasion by increasing cell adhesion, thereby blocking cell extrusion. During invasion, *Shigella flexneri* translocates effector proteins (OspE1 and OspE2) by type-III secretion system (T3SS) to the host cell cytoplasm [30]. Once entering the host cell, OspE binds to integrin-linked kinase (ILK) at the cell basement membrane; this interaction increases the level of β1 integrin at the cell surface whilst reducing the phosphorylation of focal adhesion kinase (FAK) and paxillin, which ultimately promotes cell attachment and inhibits cell extrusion [30]. *Escherichia* *coli* has a similar but even more sophisticated mechanism with which to take advantage of cell extrusion. On one hand, it releases EspO1-1 to bind to ILK, following the same mechanism as OspE [31]. On the other hand, it releases EspO1-2 to interact with EspM2 (RhoA guanine nucleotide exchange factor), which results in the inhibition of EspM2-mediated RhoA activity, accordingly suppressing actin stress fiber and cell contraction, hence blocking cell shedding [32]. 

In general, exploiting intestinal cell shedding has been used as an effective tool for many pathogens to take over the epithelial fence function and invade the body. Thus, intensive research about the role of cell shedding during invasion might help us to develop strategies against those infections.

### 2.2. Cancer

The whole homeostatic turnover, and in particular apical extrusion, might act as a tumor suppressor mechanism, by which cells carrying oncogenic mutations are rapidly eliminated from the body and eventually die due to the loss of survival signals (anoikis). In this context, the direction of cell extrusion should be regulated in order to prevent basal extrusion, which permits mutated cells to access underlying tissues, and even migrate to other sites. Simultaneously, cells driven by oncogenic mutations might acquire the ability to keep the activation of survival signals and thereby enhance their ability to invade other sites and promote tumorigenesis. Together, defects in apical cell extrusion and/or basal extrusion can be exploited by cancer cells as an effective mechanism to create cell masses, invade, and metastasize [33,34]. Actually, frequently found mutations in cancer lead to cell extrusion alterations, such as the shift in the direction of extrusion.

Adenomatous polyposis coli (APC) mutation is an early event during the initiation of colorectal cancers (CRC). APC inactivation subsequently causes the activation of the Wnt signaling pathway via β-catenin nuclear translocation, which promotes colon tumorigenesis [35]. Since cell extrusion requires actin and myosin II contraction of surrounding cells, it is not surprising that the loss of APC causes altered cell extrusion. Thus, the majority of CRCs carry APC truncations lack of EB1 and PDZ domains at C-terminus, which are also microtubule-binding domains and allow microtubule estabilization [36,37,38]. APC mutations lead to uncontrolled microtubule orientation, impeding S1P trafficking and promoting basal extrusion [39]. Marshall et al. had observed the shifting of APC in human bronchial epithelial (HBE) during cell shedding. Typical APC expression near the apical cell surface (at non-extruding state) shifted to the basolateral surface during apical extrusion, but remained at the apical surface during basal extrusion. In both cases, APC localized in close proximity to actomyosin contraction sites. Moreover, in HBE cell monolayer and zebrafish epidermis, cells lacking APC or carrying oncogenic APC mutations preferred to extrude basally instead of apically [40]. Interestingly, even though the extruding cell is squeezed out via actin/myosin contraction of neighboring cells, APC regulates this contraction by controlling microtubule and actin in the dying cell itself [40]. Putting this all together, investigating the function of APC-dependent microtubule-stabilization and the control of cell shedding direction might help us to understand colon cancer progression and to prevent its invasiveness. 

Another oncogenic mutation that is associated with cell shedding alterations is K-RAS, which was found in 31% of CRCs [41]. Cells carrying oncogenic K-Ras depicted downregulated expression of S1P and its receptor S1P2 due the activation of autophagy [34,42]. Thus, strongly elevated survival signals promoted cell/tumor growth, while autophagy-mediated S1P degradation drove cell to extrude basally. Accordingly, inhibiting autophagy in the K-Ras^v12^ cell rescued the S1P level and apical extrusion [34]. A recent study identified a new mechanism by which K-RAS mutated cells control cell plasticity and promote basal extrusion. Transformed epithelial cells mechanically pinch off the epithelial cell surface determinants (apical markers, such as E-cadherin and ZO-1), which enables their plasticity. Although K-RAS mutation alone can drive basal extrusion, many basal-extruded cells died within a few days due to the activation of p53, a pro-apoptotic gene frequently mutated in K-RAS-driven cancers. Accordingly, K-RAS-mutated cells lacking functional p53 are able to survive in the zebrafish body; thus, upon cell division, this leads to internal cell mass formation, migration into the bloodstream, and differentiation into stromal, neuronal-like, and other cell types [43]. This study indicates new insight that could account for the intrinsic metastatic potential of K-RAS-driven tumors. Understanding the mechanism of how oncogenic cells invade the underlying tissue, particularly in case of hijacking cell extrusion, might help in the development of new treatment modalities in metastatic disease.

### 2.3. Inflammation

In physiological conditions, a shed cell will leave a gap or discontinuity in the epithelium, which is quickly covered by TJ-resealing in the neighboring cells and is fulfilled by new cells generated from crypts. In inflammatory conditions, however, excessive shedding rate and failure of TJs redistribution implicate that cell loss cannot be compensated, potentially leading to focal permeability defects and epithelial gaps, as seen in Inflammatory Bowel Disease (IBD) [44,45,46]. IBD is a chronic intestinal disorder exemplified by Ulcerative Colitis (UC) and Crohn’s disease (CD). Common IBD symptoms are diarrhea, abdominal cramps, weight loss, bloating, and blood in stool. The underlying causes of IBD are not fully understood, but genetics, exacerbated immune response, and dysbiosis are thought to be largely involved. Besides this, many external factors can increase the risk of developing IBD, such as age, smoking, stress, or diet. IBD in some cases leads to serious life-threatening complications such as colitis-associated neoplasias and cancer [47]. At present, IBD has become a global disease which affects approximately 0.5% of North American and European populations [48], and is increasing in newly industrialized countries in Africa, Asia, and South America [49]. Besides well-established but unspecific IBD treatments (anti-inflammatory, immunosuppressive, and biological therapy), surgery (colectomy) is in some cases required as the last therapy option in UC patients. Indeed, since many patients do not respond to available drugs and causative treatment is still not available [50,51], numerous new therapeutic approaches are currently being tested, such as cytokine inhibitors (anti-IL-6, -6R, -12, -23, or apermilast), molecules interfering with cytokine mediators (JAK inhibitors, SMAD7 blockers), blockers of transcription factors (GATA3, ROTɣt), and agents targeting T-cell trafficking (integrin blockers, anti-MAdCAM1). This highly emphasizes the need of intensive research in the field to improve our knowledge and provide innovative treatment approaches in IBD.

It has been suggested that epithelial gaps could be exploited as a prediction tool for hospitalization and surgery in IBD patients [52]. In humans, the detection of epithelial gaps by confocal laser endomicroscopy (CLE) upon fluorescein administration might be utilized as a method via which to evaluate the mucosa and assess the inflammation based on epithelial alterations [53]. Therefore, Kiesslich et al. had established the so-called “Watson score” to evaluate the severity of barrier dysfunction based on cell shedding in vivo. In this endomicroscopic grade, “normal” status (grade I) is defined as only single cells being shed without any barrier defect. “Functional defect” (grade II) is defined when a single cell is being shed, but visible leaked fluorescein in the lumen is detected. If multiple cells per site are being shed, it is designated as a “structural defect” (grade III). The detachment of multiple adjacent cells exposes the lamina propria into the lumen, so-called microerosion, leading to a large breach in the epithelium, which cannot be sealed and causes barrier loss [44]. Taking advantage of the mentioned score, the authors examined non-inflamed/damaged colon or terminal ileum areas from IBD patients, and figured out that there were considerably more epithelial gaps, microerosions, and fluorescein leakage in IBD than control patients. After the endomicroscopic examination, the patients were prospectively followed, and 24 out of 58 patients suffered from a flare of their diseases within a year. Using the Kaplan-Meier analysis to assess the relapse following stratification by their Watson scores, they observed that patients suffering from a flare showed remarkable higher occurrence of fluorescein leakage and microerosions, with higher Watson grade than patients whom did not have a flare. Taking advantage of CLE, another group studied terminal ileum of 16 CD and 12 UC patients, and showed that inflamed tissues had significantly higher median epithelial gap densities, but no correlation between gap density and disease activity was noted [54]. It is not clear whether the failure of intestinal barrier leads to inflammation or vice versa, but many rodent studies have shown that maintaining intestinal barrier integrity is undeniably important in directing episodes of intestinal diseases. Transgenic mice with increased TJ permeability exhibited significant hallmarks of IBD and suffered from a more rapid and severe inflammation upon adoptive T cell transfer, due to aberrant subclinical mucosal immune activation [55]. 

Being the primary digestive organ, the intestine serves two main functions: nutrition/absorption and restricting the transfer of harmful luminal pathogens into the circulation; hence, it represents the first immune barrier of the body. Considering its structure, the mucosa is the intestinal outermost layer facing the gut lumen, and it is represented by a layer of intestinal epithelial cells (IECs) and the lamina propria—a subepithelial connective tissue containing various infiltrating immune cells. The epithelial monolayer is a key element of the intestinal barrier; it is composed of specialized and polarized cells connected via intercellular junctions, including TJs, adherens junctions (AJs), and desmosomes, permitting the sealing against the environment. When this barrier is compromised, microbes present in the gut lumen can penetrate and activate immune cells. Thus, exacerbated immune reactions against the intestinal microbiota can trigger local inflammation, which might result in the development of gastrointestinal disorders [56]. Barrier function alterations featured by increased intestinal permeability (or “leaky gut”) is reported and well described in IBD patients [57]. Thus, epithelial alterations are nowadays considered to participate in the onset as well as in the promotion of inflammation in the context of IBD. Strikingly, IBD patients in remission and IBD patient relatives demonstrate increased intestinal permeability before flares [58] or diagnosis [59,60,61,62,63], respectively. This claims for the occurrence of epithelial alterations before the outbreak of the inflammatory response, and therefore suggests epithelial disruption as an etiological factor in IBD pathogenesis. In agreement, several animal models demonstrate that epithelial permeability precedes the development of colitis [64,65,66], while patients suffering from monogenic diseases affecting genes encoding for epithelial proteins develop IBD-like phenotypes [67,68]. However, the low phenotype penetrance of these monogenic diseases supports the hypothesis that a leaky barrier needs an additional trigger for the development of intestinal inflammation. Thus, JAM-a deficient animals as well as claudin-2-expressing transgenic mice have a leaky barrier but no colitis [69,70]. It´s a matter of discussion within the scientific community whether epithelial intrinsic alterations, occurring in the absence of mediators derived from other cell types, can represent declutching factors for intestinal inflammation. Further investigations are needed in order to decipher the causative role of epithelial alterations in IBD.

In conclusion, intestinal barrier integrity highly depends on cell extrusion. The fact that excessive or defective cell extrusion could lead to multiple intestinal diseases emphasizes the need for more studies about involved mechanisms and factors that control cell shedding in the context of intestinal homeostasis. In this review, we provide an overview of these mechanisms (see Section 3, Section 4, Section 5, Section 6 and Section 7). Likewise, further studies should also focus on the development of drugs that target and/or control cell shedding, and thereby promote mucosal healing.

## 3. Mechanosensations

Even though apoptotic cell shedding has been thoroughly studied, mice lacking key proteins involved in programmed cell death showed no abnormal cell shedding (details in Section 4). In agreement with this, in 2012, Rosenblatt et al. showed that a high number of cells undergoing spontaneous extrusions in human colon and zebrafish epidermis showed no signs of apoptosis [12]. Strikingly, cells were eliminated broadly at areas of high cell density, such as in the colonic surface or zebrafish fin edges. This study drew researchers’ attention to non-apoptotic shedding mechanisms, since apoptosis-cell shedding has been defined as not essential for extrusion process during homeostasis. These observations implicated that live rather than dead cell extrusion dominates epithelial turnover for maintaining tissue homeostasis and development. Seeking potential pathways involved in cell extrusion, they took advantage of MDCK cells and focused their attention on stretch-activated channels. Stretched-activated or mechanosenstive channels are important for almost every organism, as most cellular processes (volume regulation, migration, and differentiation) require mechanosensations. Mechanosensitive channels are responsible for converting mechanical stimuli from the environment into electrochemical activities to drive diverse cellular processes, including feeling skin contact, gravity, proprioception, sound waves, food texture, muscle stretch, and air flow [71]. Among mechanosensitive channels, the Piezo family (meaning “pressure” in Greek), were discovered in 2010 [72] and have been a breakthrough in mechanotransduction research in the last decade. Most vertebrates contain two Piezo paralogs (namely Piezo1 and Piezo2), which play key roles in a wide range of physiological processes. Recently, David Julius and Ardem Patapoutian unlocked one of nature’s secrets by uncover the role of Piezo channels in the human perception of touch and proprioception, explaining at the molecular basis our ability for sensing heat, cold, and mechanical force (The 2021 Nobel Prize in Physiology or Medicine). Focusing on epithelial homeostasis, blocking Piezo1 by gadolinium (Gd3^+^) or knocking down Piezo1 leads to cell shedding blockade in zebrafish, and this can trigger the development of epidermal cell masses in the fin’s edges where migrating cells have failed to eject [12]. Since the accumulation of S1P–a key signal for apical extrusion–only occurs shortly before extrusion in MDCK cells, S1P is likely a downstream target of Piezo1. However, studies deciphering the functions of Piezo1 upstream of the S1P-S1P2-Rho pathway in mammalian intestine are still limited. Based on this interesting study, it is now well established that there are two types of cell shedding characterized by the activation of caspase-3 (apoptotic) and live or Piezo1-dependent shedding, which plays the leading role in homeostasis. In a follow-up story, Rosenblatt et al. showed that Piezo1 is required for both cell division and extrusion processes in MDCK cells. They proposed a model where Piezo1 acts as a paradoxical signal, controlling cell numbers by sensing crowding and stretching. Accordingly, Piezo1 expression shifts from the nuclear envelope (during cell division—stretching) to the cytoplasm/plasma membrane, and in the end accumulates over time in the cytosol (during cell extrusion—crowding) [73]. These results suggested that Piezo1 localization and expression might determine the outcome upon sensing the mechanical input, and thereby adjusting cell numbers to reach steady state or homeostatic conditions. Since it has been shown that Piezo1 is associated with both cell division and extrusion in vitro/zebrafish, it is now an open question as to whether it could act as a dual-sensor regulating intestinal epithelial number in mammals, by triggering cell division at the crypt bottom and cell extrusion at the villus tip.

Mechanosensations control tissue growth and repair after injury. During growth, tissue mechanics is defined by epithelial cell packing within neighboring cells. Thus, low density results in the lamellipodia-based crawling and collective cell migration, while increased cell density promotes acto-myosin contraction of neighboring cells and cell extrusion [74]. Interestingly, these two phenomena are significantly dependent on RHOA/RAC1-mediated cytoskeleton dynamics (see VIa). Eisenhoffer et al. provided new evidence on how stretch-activated ion channels affect cellular behavior after tissue injury [75]. Taking advantage of high-resolution time-lapse imaging techniques, the authors observed that wound-induced collective cell movements increase cell numbers at the wound edges, hence promoting the elimination of damaged or aged cells and enhancing tissue repair. To examine the role of mechanical forces in the wound healing process, amputated zebrafish larval tissues were treated with gadolinium or GsMTx4 (mechanosensitive channel inhibitors) to disrupt stretch-activated ion channels. Strikingly, cells failed to extrude after chemical perturbation, consequently causing cell accumulation and increased wound area size. These data firmly emphasized the essential function of stretch-activated ion channels in cell extrusion that may contribute to epithelial tissue maintenance and regeneration [75]. Of interest, Piezo1 protein was also found to be involved in keratinocyte migration and wound closure [76]. Accordingly, mice lacking epidermal Piezo1 had faster wound closure speed, and epidermal Piezo1 gain-of-function mice decelerated wound healing process. Moreover, in vitro studies in isolated murine keratinocytes suggested that Piezo1 activity slowed down keratinocyte movement. Piezo1 subcellular localization determined the re-epithelization, i.e., enriched Piezo1 areas along the wound margin resulted in wound edge retraction and delay of wound closure. These findings imply a potential therapeutic use of Piezo1 inhibition in accelerating wound recovery. However, considering the key roles of Piezo1 in sensory afferent firing and behavioral response in keratinocytes [77], the quality of wound healing in the absence of Piezo1 might require an in-depth evaluation [76]. 

## 4. Cell Death

In the context of rapid renewal of the intestinal epithelium, cell death is not only important for the extrusion of aged or damaged cells, but also essential to eliminate infected cells as a self-protective mechanism. Cell death is seldomly detected along the villus and more frequently at the tip where cells are lining the lumen [78], probably due to its link to cell shedding. Little, if anything, is known about physiological cell death at the crypts; however, it appears to be a predominant target under pathological conditions [79,80]. Dysregulated cell death is a hallmark of intestinal inflammation that has been detected in IBD patients and preclinical colitis models [80,81,82]. There are several types of cell death depending on the triggering stimulus and subsequent cellular signaling pathways, designated as apoptosis/anoikis, necrosis, necroptosis, pyroptosis, and ferroptosis, among others (Table 1).

### 4.1. Apoptosis

Apoptotic cell death is recognized by cell shrinkage, chromatin condensation, membrane blebbing, and caspase activation. Elevated apoptosis rate had been noticed in colonic LP specimens of UC patients [91], and also in patients requiring surgery compared to those whose disease activity can be controlled with medication [92]. Also supporting the contribution of programmed cell death in IBD, the apoptosis-inducing factor (PDCD8) was identified as the most regulated molecule in a proteomic analyses from intestinal tissue from IBD patients [93], along with the upregulation of PUMA (p53-upregulated modulator of apoptosis) in UC patients, which was associated with the degree of apoptosis induction and colitis severity [81]. As mentioned above, cell death (dependent cell shedding) is intimately connected to apoptosis. Already in 2011, Rosenblatt´s group demonstrated that apoptotic cell extrusion requires the action of caspases [85]. During homeostasis, extruding cells undergo a detachment-dependent apoptotic cell death, as their basal membrane is released from the extracellular matrix. This process is particularly referred to as anoikis. In this process, apoptosis is initiated by the activation of caspase-2 and -9, followed by the hierarchical activation of downstream caspases such as caspase-3 [83]. It is controversial whether cell apoptosis and caspase activation promotes cell detachment leading to cell shedding, or they are indeed the results of cell shedding. Ghazavi et al. has confirmed that mice lacking both caspase-3 and caspase-7 in IEC showed neither morphological/inflammatory alteration nor intestinal dysbiosis at steady state [94]. This was explained by the compensation of increased non-apoptosis cell shedding in the absence of executioner caspases, likely through the mechanical force exerted by surrounding cells, since there was no alteration regarding other cell death mechanisms. These studies suggest that cell apoptosis is maybe a consequence of cell shedding, and thus is dispensable for the maintenance of epithelial integrity.

Cell shedding at the villus tip can be initiated by various apoptosis triggers, for example, tumor necrosis factor (TNF) or ischemia, but surprisingly not by genotoxic injury like radiation [14]. Back in 2006, Bullen et al. had observed that although IECs always underwent apoptosis during cell shedding, apoptotic bodies (apoptotic fragments) were never detected in the monolayer, even though cleaved cytokeratin 18 or cleaved caspase-3 positive cells were frequently detected at the villus tips [84]. Strikingly, Marchiando at el. could visualize cell shedding events before the signal of caspase-3 was detected. Notably, Rho-associated kinase (ROCK) is cleaved and activated by caspase-3, which contributes to myosin light chain (MLC) phosphorylation and is needed for membrane blebbing during apoptosis [95,96]. Moreover, blocking caspase by wide-spectrum caspase inhibitor did not affect MLC phosphorylation, suggesting that caspase-3 is activated after the initiation of cell shedding, and that TNF-induced cell shedding is associated to apoptosis rather than anoikis [18]. Moreover, in case of TNF-induced extrusion, cleaved caspase-3 positive cells are found fully integrated with other cells in the epithelial layer. This observation demonstrated that apoptosis may occurs as a primary event that enhances cell detachment, hence leading to cell shedding, at least in a pathological context.

In contrast to apoptosis, necrosis is a premature kind of cell death induced by complex signal transduction pathways and execution mechanisms [97]. Necrosis is recognized by cell rounding, cytoplasmic swelling, dilated organelles, and chromatin condensation [98]. In contrast to apoptosis, necrotic cells are not eliminated by cell extrusion, but rather by passive mechanisms [85]. 

### 4.2. Necroptosis

Necroptosis is another form of programmed cell death, which shares common morphological features with necrosis but is regulated by the serine–threonine kinase receptor-interacting protein RIP [99,100,101]. It is featured by the trafficking and accumulation of the mixed linked kinase-like protein (MLKL) from necrosome to the plasma membrane [102]. In fact, necroptosis also contributes to a broad range of pathological cell death events, including IBD [82,103]. Mice lacking IEC-Fas-associated death domain (FADD), an adaptor protein of death-receptor-induced apoptosis, developed spontaneous epithelial necrosis, lack of Paneth cells, enteritis, and severe erosive colitis. Genetic deficiency of RIPK3 could rescue these phenotypes, suggesting that intestinal colitis was initiated by RIP3-dependent death of FADD knockout mice [104]. Likewise, mice carrying caspase-8-deficient intestinal epithelium developed spontaneous ileitis due to RIP3-dependent paneth cell necroptosis [103]. Moreover, mice lacking SETDB1 in Intestinal Stem Cells (ISCs) developed enteritis, which could not be rescued by IEC-caspase-8 knockdown or caspase inhibitor (Z-VAD-fmk), but by MLKL or RIPK3 deletion, indicating that targeting necroptosis specifically in ISCs may represent a new approach for IBD treatment [82]. Of note, the accumulation of phosphorylated MLKL at intercellular junctions, which facilitates necroptosis of neighboring cells, is thought to be relevant in the context of IBD [102]. Together, these studies confirmed the important role of RIPK3-mediated necroptosis in maintaining epithelial homeostasis, and suggested that necroptosis inhibition might protect against IBD.

### 4.3. Pyroptosis

Pyroptosis is a programmed and inflammatory cell death form, which should be considered an important immune defense mechanism, associated with pathological cell shedding. Pyroptotic cells also show chromatin condensation and DNA fragmentation as in the case of apoptosis, but their nuclei stay intact. Mediated by several inflammasomes through a caspase-dependent cascade, pyroptosis leads to the separation of GSDMD N- and C-terminals, and subsequent membrane pore formation. While apoptosis is triggered by caspase-2, -8, -9, -10, and effector caspase-3, -6, -7, pyroptosis is triggered by caspase-1, -4, -5, and -11 [105]. In IECs, NAIP-NLRC4 inflammasome activation led to IL-18 and eicosanoid release, resulting in cell death accompanied by membrane permeability and rapid IECs extrusion. This sufficiently protected mice against *Salmonella* invasion, but also caused certain pathology. Surprisingly, IECs extrusion did not necessarily require caspase-1 or gasdermin D, as caspase-8 inflammasome activation could compensate the loss of caspase-1 [89]. A recent study taking advantage of CLE had shown that the distal duodenum of functional dyspepsia patients had dramatically increased numbers of epithelial gaps (cell extrusion) compared with the heathy control, correlating with impaired epithelial integrity and increased number of IECs undergoing pyroptosis [106]. Moreover, pyroptosis-associated cell shedding was detected in the murine Salmonella enterocolitis model, where *Salmonella Typhimurium* (*S. Typhimurium*) invaded intestinal enterocytes and expanded in *Salmonella*-containing vacuoles (SCV), causing intraepithelial microcolony formation, thus activating NAIP/NLRC4 inflammasome and caspase-1 at an early stage. Afterwards, *Salmonella* hyperreplication occurred in both SCV and cell cytosol, and cytosolic LPS then activated caspase-11 via the non-canonical inflammasome pathway. The activation of either caspase-1 or caspase-11 eventually led to pyroptotic death and subsequent extrusion of infected cells. Moreover, the activation of inflammasomes also induced cytokine secretion, triggered immune cell recruitment and mucosal inflammation [87,88]. This defense mechanism allowed the body to get rid itself of infected enterocytes and restrict *Salmonella* expansion. Considering NEK7 as an essential downstream protein of NLRP3 activation, Chen et al. found out that intestinal epithelial cells lacking NEK7 abolished ATP-LPS-induced pyroptosis, and NEK7-deficient mice exhibited milder colonic erosions and edema than control mice, as well as ameliorated DSS-induced colitis. These data suggested a mechanism in which NEK7—NLRP3 interaction controls inflammasome activation and pyroptotic cell death, hence contributing to inflammation development via NFκB signaling [107]. In addition, other studies also suggested that pyroptosis plays a crucial role in gut immune defense by controlling microbial infections, IL-18, ROS secretions, potassium efflux, or lysosomal damage [108]. A newly released publication investigating the effect of TNFα on different mouse models has shown that mice lacking interferon regulatory factor 1 (IRF1) and caspase-3 were fully protected upon TNFα stimulation. Interestingly, IECs from control mice depicted disruption of membrane integrity, swelling, and GSDME-N terminal fragments, implicating pyroptosis induction. These features were reversed in GSDME knockdown mice, and required the contribution of IRF1-mediated caspase-3 [90]. The authors also found that IFR1 protein levels vastly increased in IBD patients, suggesting that IRF1 might have a pathogenic role in IBD, and blocking the positive feedback loop between IRF1 and TNF might be beneficial for IBD cure [90]. In summary, pyroptosis is a unique inflammation-related cell death, highly associated with cell shedding.

### 4.4. Ferroptosis

Ferroptosis is a regulated necrosis-related cell death, triggered by overwhelming iron and lipid oxidation. This recently recognized cell death pathway has typical morphological traits of mitochondrial shrinkage, increased mitochondrial membrane density, and disappearance of mitochondrial cristae. Ferroptosis is induced by iron accumulation and lethal reactive oxygen species (ROS) release, via the Fenton reaction, glutathione (GSH) depletion, and/or inactivation of glutathione peroxidase 4 (GPX4) [109,110]. Even though it was reported to be associated with intestinal epithelium dysfunction, there are limited studies about the role of ferroptosis in intestinal diseases [111]. In mice treated with DSS, ferroptosis was found to be induced, demonstrated by iron overload, GSH attenuation, ROS and MDA production, and decreased expression of SOD and GXP4. These altered features of ferroptosis could be reversed by an anti-inflammatory substance named Curculigoside (CUR), potentially via regulating GXP4 levels [112]. In agreement with this, other study also showed that blocking ferroptosis via NRF2/HO-1 signaling pathway was able to ameliorate severe symptoms of DSS-induced colitis in mice, and suggested ferroptosis inhibition as a potential therapeutic target for UC [113]. Moreover, a recent study in DSS-treated mice also emphasized the importance of ferroptosis in response to anti-colitis and anti-carcinogenesis of OPSSP167 activity [114]. Last but not least, the most recent study suggested that administration of Ferrostatin-1 (ferroptosis inhibitor) could improve symptoms of TNBS-induced colitis mice, and thereby identified a new therapeutic approach in CD treatment [115]. In human IBD, ferroptosis was known to contribute to UC via ER stress-mediated IEC cell death, which could be relieved by NF-κBp65 phosphorylation [116]. Regarding cancer, the function of ferroptosis in CRC was mainly focused on the activity of TP53 protein—a ferroptosis regulator and tumor suppressor. TP53 limits erastin-induced ferroptosis by blocking dipeptidyl-peptidase-4 (DPP4) activity, while silencing TP53 results in increased ferroptosis in CRC cells and enhancing the anti-tumor activity in mouse models [117]. Accordingly, enhancing TP53 activity/ferroptosis appears to be a highly desirable target for colon cancer therapy [118,119,120]. Depending on the inflammatory/tumorigenesis context, ferroptosis acts as positive or negative regulator, and this dual regulatory roles was already discussed in detail in a recent review [111]. Although studies about the function of ferroptosis in intestinal disease are still at an early stage, they provided us with new insight of ferroptosis function in the gut in the context of IBD pathogenesis and treatment, similarly also highlighting the need of further investigations. However, so far there is no description of an association between ferroptosis and cell shedding.

Considering that cell survival/death is important in IBD and IBD-associated complications such as CRCs, cell death-based therapeutic treatment could plausibly bring beneficial effects to IBD treatment. However, it could be a two-edged sword: inducing cell death or cell shedding via cell death pathways may be beneficial for tumorigenesis, while inhibiting excessive cell death/shedding may promote healing upon inflammation.

## 5. S1P Pathway

S1P is an important membrane-derived lysophospholipid signaling molecule, implicated in various cellular processes, like cell proliferation, migration, and cytoskeleton rearrangement. Thus, the S1P pathway plays a key role in inflammation and immunity [121]. S1P intracellular levels are regulated by several metabolic steps. Produced by two sphingosine kinase isoenzymes, SphK1 and SphK2, S1P can be degraded via S1P lyase (SGPL1) or dephosphorylated by S1P phosphohydrolase 1 and 2 (SGPPP1/2) or lipid phosphate phosphatase 3 (PLPP3). These metabolic processes maintain low intracellular levels of S1P in most tissues. Alterations of tissue homeostasis, such as those occurring upon inflammation or in cancer, allow the accumulation of S1P leading to the activation of S1P-dependent cellular machinery [122]. Moreover, S1P exerts autocrine and paracrine functions through G protein-coupled receptors (SP1R1-5). Elevated levels of S1P produced by red blood and endothelial cells as well as location-specific functions of metabolic enzymes, transporters, and chaperones contribute to S1P circulatory/tissue gradient. Thus, elevated S1P levels are found in blood and lymph, in comparison to most tissues. In fact, this gradient is a key mechanism for S1P-mediated control of cell trafficking [123], and vascular tone and integrity [124]. Like intracellular S1P levels, concentration gradients are also altered in pathological conditions, and thereby might contribute to the disease. Together, S1P levels and signaling are critical to the control of several actions in the immune system. Beyond immune system-related actions, S1P play a key role in cardiovascular and central nervous systems. 

Previous data have shown that apoptotic cells as well as live shedding cells produce S1P as a communication tool between the shedding cell and its neighbors. Recent data also revealed that S1P synthesized by SphK2 and not SphK1 of the dying cell itself is crucial for initiating apoptotic cell extrusion; blocking or knocking down SphK2 in MDCK cells lead to cell-cell junction degradation and block cell shedding [125]. In Jurkat T cells, caspase cleavage of SphK2 [126] or induced SphK1 expression [127] have been suggested as triggering mechanisms for S1P release from apoptotic cells. Although this can also occurs in the case of apoptotic IECs, still unknown are the triggering mechanisms in the case of Piezo1 activation in live cell extrusion. Released S1P then activates the S1P2 receptor of surrounding cells, leading to a RhoA/p115RhoGEF-dependent actomyosin basolateral contraction of those cells, and hence mediates apical cell extrusion [128,129,130]. Tension-sensitive mechanotransduction determines the activation of RhoA in the immediate neighboring cells; but this mechanotransduction requires the S1P-S1PR2-dependent accumulation of Gα12/13 in the epithelium, which is needed for E-cadherin and Myosin VI-dependent p115 RhoGEF activation. So S1P and mechanotransduction might act simultaneously to activate acto-myosin contraction in non-shedding cells, which permits cell shedding completion. Thus, this only occurs in neighboring cells which are exposed to the released S1P and are also able to sense the hypercontractility of the apoptotic cell [131].

Demonstrating that it is indispensable for cell extrusion, blocking of S1P signaling in MDCK cells leads to the retention of apoptotic cells in the monolayer. Moreover, zebrafish carrying S1P2 loss-of-function mutations showed a significantly higher percentage of non-extruded apoptotic epidermal cells [128]. These data implicated that S1P signaling is mandatory for epithelial cell extrusion and may be involved in driving stem cell delamination during differentiation or tumor invasion. Alteration of S1P, S1P2 receptor, p115RhoGEF, or actin/myosin contraction can inhibit apoptotic cell shedding and create gaps in the epithelia [128,130]. However, disruption of these pathways do not affect basal extrusion, implicating that in a situation where apical extrusion is impaired, the occurrence of basal extrusion might in turn contribute to tumorigenesis, invasiveness, and metastasis. 

As already mentioned above, K-RAS mutation could drive cells to extrude basally due to the loss of cellular apical markers together with the downregulation of S1P/S1P2 [34]. Highlighting the relevance of S1P-dependent apical extrusion in cancer, lowering S1P2 levels in human bronchial epithelial (HBE) or zebrafish caused apical extrusion defects and apoptosis resistant cell mass formation [132]. Cells with disrupted extrusion also became more resistant to chemotherapy, and interestingly, boosting exogenous S1P2 expression helped to rescue these phenotypes as well as to reduce orthotopic pancreatic tumor size and metastases in mice [132]. Together, this could explain why some carcinomas expressing little to no S1P2, like pancreatic ductal adenocarcinomas [133] or lung tumors [134], are generally more aggressive and more challenging to treat. In fact, simultaneous loss of p120 catenin and Kras activation resulted in basal extrusion, due to NFkB-dependent impaired expression of S1P signaling related genes. In this case, basally extruded cells are still viable and possess invasion potential. Thus, those represent early events in metastasis and invasive pancreatic neoplasia [135]. Considering the polarity of epithelial cells, restricted apical extrusion is regulated by the S1P-Rho pathway [128]. Contemplated as an intrinsic epithelial mechanism of anti-tumor activity, cell competition between normal and newly transformed single cells contributes to the elimination of potentially harmful cells (EDAC). Interestingly, knock-out of S1PR2, specifically in normal cells, prevents apical extrusion of neighboring RasV12-transformed cells. Mechanistically, the paracrine effect of S1P promotes the RhoA-dependent accumulation of filamin in normal cells, which in turn promotes EDAC [136].

In summary, alterations of the S1P pathway might lead to basal extrusion and hinder epithelial intrinsic mechanisms to eliminate transformed cells, which contributes to tumor initiation and progression.

## 6. Cytoskeleton Dynamics

Regulated cytoskeleton function and control of cell polarity is crucial for epithelial barrier function and adequate cell extrusion. Cell cytoskeleton comprises of microfilaments (actin), intermediate filaments, and microtubules (tubulin) within the cellular cytoplasm. This dynamic filamentous structure is critical for maintaining cell shape, cell mechanical deformation resistance, and tissue architecture, and is thereby involved in numerous cellular functions like molecule transport, cell division, etc. Accordingly, recent data implicate the cytoskeleton within epithelial cells as a critical regulator of the mucosal barrier under physiological and pathophysiological conditions. For instance, Alix-mediated assembly of the actomyosin-tight junction polarity complex preserves epithelial polarity and the epithelial barrier [137]. Mice with an epithelial-specific knockout of NMiiA present increased intestinal permeability and low-scale mucosal inflammation (although they do not develop spontaneous colitis) and increased susceptibility to experimental colitis [138]. Cell stress-induced changes in actin dynamics affect actin-binding proteins, such as Villin-1 and Gelsolin, which in turn control the survival of IEC and barrier function [139]. ACF7, a crosslinker of microtubules and F-actin, regulates intestinal wound repair by orchestrating tight junction dynamics. ACF7-conditional KO mice in IECs were more susceptible to DSS-induced colitis, but they developed no spontaneous inflammation [140]. Accordingly, reduced ACF7 expression correlated with the development of UC in humans. Together, several mouse studies demonstrated that alterations of the cytoskeleton resulted and/or contributed to intestinal inflammation, suggesting that intestinal barrier defect, represented by cytoskeleton dysregulation, might contribute to IBD pathogenesis.

### 6.1. Rho GTPases

Rho-GTPases are known as important molecules regulating cytoskeletal reorganization. Cycling between GDP- (off state) and GTP-bound (on state) forms, activated RhoGTPases bind to different downstream protein kinases and actin-binding proteins, resulting in the local assembly or disassembly of filamentous (F)-actin [141], hence regulating cytoskeleton remodeling. For instance, RhoA activation leads to Rho-associated kinase (ROCK)-dependent MLC phosphorylation, facilitating myosin binding to actin and promoting contractility. Notably, ROCK is cleaved and activated by caspase-3, which contributes to myosin light chain (MLC) phosphorylation and is needed for membrane blebbing during apoptosis [95,96]. The function of Rho proteins in different IEC subtypes in the context of intestinal inflammation/cancer had been summarized in a recent review [51]. Upon S1P2 activation on neighboring cells [128], cell shedding is executed via Rho-dependent acto-myosin contractility and redistribution of intercellular junction proteins. In fact, over half of the intestinal cell shedding events has been associated with phosphorylated MLC [84].

Perhaps the most studied Rho protein, RhoA is a master regulator of cytoskeleton in different cell types. In the context of intestinal inflammation, controversial studies suggest that accurate RhoA function is essential for intestinal homeostasis, and both RhoA inhibition and hyperactivation cause gastrointestinal alterations. Thus, it has been described that ROCK is highly activated in the inflamed intestinal mucosa of patients with CD [142]. However, our two previous publications linked RhoA inhibition within intestinal epithelium as well as T cells to intestinal inflammation, by controlling epithelial barrier function [143] and T cell trafficking [144], respectively. Interestingly, mice lacking RhoA or prenylation machinery in IECs exhibited instability of IEC shape and accumulation of arrested cell shedding events, defined by the appearance of funnel-like structure of actin fibers, consistent with increased gap formation and the breakdown of intestinal barrier function [143]. As mentioned above, several publications demonstrate that RhoA is an important mediator in epithelial cell extrusion, since it mediates actin-myosin contractility leading finally to cell extrusion. Accordingly, blocking Rho or Rho kinase completely blocks both apoptotic and non-apoptotic cell ejection [12,18,129], implicating that removal of both apoptotic or live cells depends on non-autonomous Rho signalling and multicellular actomyosin ring assembly. In pathological cell shedding, apoptotic cells require autonomous actomyosin force provided by an actomyosin ring formed in the dying cell, which leads to the formation of rosettes around apoptotic cells in neighbouring cells prior to the assembly of the multicellular actomyosin ring [15,129]. Furthermore, the activation of Rho-mediated actin-myosin assembly and contraction via p115RhoGEF is also relevant for the direction of cell extrusion [130]. As mentioned above, bacteria also exploits RhoA function to hijack cell shedding, as seen in Escherichia coli infection [32]. Taking into account that the recruitment of GTPases is coordinated by post-translational prenylation, targeting prenylation has emerged as a prospective candidate in the context of epithelial integrity and gut homeostasis. Besides RhoA, Rac1 and Cdc42 also have profound effects on regulating intestinal homeostasis. Mice carrying Cdc42-deficient IEC showed signs of severe intestinal alterations, featured by large intracellular vacuolar structures containing microvilli, defective cell proliferation and differentiation [145], and/or intestinal hyperplasia, impaired epithelial polarity, proliferation, differentiation, and migration [146]. Rac1 was also shown to be implicated in the import and intracellular processing of signals that mediate intestinal epithelium differentiation [147], and to be involved in intestinal cell proliferation [148] and migration [149,150]. Moreover, the atypical RhoGTPase RhoU was found to be reduced in human colorectal tumor tissues, and this correlated with an increase in RhoA activity coupled with the change of phosphorylated MLC2 level, which triggers actomyosin contraction and ultimately promotes cell shedding [151]. Thus, despite this well described role of RhoA in epithelial cell extrusion, little is known about other Rho GTPases in this context. Our recent publication demonstrated that Rac1-mediated cytoskeleton rearrangement controls intestinal epithelial cell shedding [152]. 

Closely connected to cell shedding, Rho, Rac, and Cdc42 play important roles in epithelial wound closure; this requires major cell shape changes as well as the equilibrium between cell movement and differentiation, which is mediated by extensive cytoskeleton remodeling and cellular junction reassembly [153]. Considering RHOA/RAC1 opposing and complementary functions, the geometry of the epithelial gap is a deciding factor for the participation of RHOA-dependent actin ring closure, or RAC1-mediated cell crawling [154]. In vitro studies have shown that IEC migration is modulated by RhoA/ROCK signaling via the activation of chemokine receptors (CXCR4 and CXCR12), and subsequent accumulation of Rho-GTP and F-actin at the leading wound edges [155]. Furthermore, RhoA/ROCK and mDia1 signaling is imperative for Caco-2 cell migration through repetitive deformation, and it is believed to play an important role in sealing the mucosal surface upon inflammation [156]. Relevantly, disrupted RhoA/ROCK1-mediated MLC phosphorylation upon myosin IXb (myo9b) depletion in Caco-2 cell resulted in faulty migration into the wound boundary, along with altered TJs localization, for example, occludin, claudin-1, and ZO-1 [157]. Likewise, a previous study had shown that the activation of Rac1 was governed by GPCR and Src-mediated signaling, resulting in ROS production via NOX1, leading to increased phosphorylation of focal adhesion proteins (FAK and Pax), which regulate collective migration of intestinal epithelium [150]. In harmony with this data, an ulterior study has shown that activated Rac1 along with increased level of ROS is essential for cell migration during intestinal wound healing in the presence of platelet activating factor (PAF), which is elevated in inflamed mucosa in both human IBD and rodent colitis models [158,159,160].

### 6.2. Intercellular Junctions

Being the key counterpart of the actin-myosin system, intercellular junctions, mediated by AJs, desmosomes, TJs, gap junctions, hemidesmosomes, and focal adhesions, stabilize cell to cell as well as cell to ECM interactions, and thereby play important roles in sealing the paracellular barrier [161]. Considering the important functions of AJs and TJs in sealing gaps during cell extrusion, it is not surprising that there is abundant evidence illustrating the relationship between these and IBD. Interference with intercellular junction proteins can originate increased intestinal permeability in vitro, such as occludin knock-down [162], and in vivo, such as JAM-A-deficient mice [163]. 

TJs characterize the boundary between the apical and basolateral membrane domain, and regulate the diffusion of solutes through the paracellullar spaces [164], therefore playing an important role in sealing the paracellular barrier. The TJ family includes both transmembrane proteins (occludin, tricellulin, claudins), junctional adhesion molecules (JAMs), and peripheral membrane proteins (zona occludens (ZO), cingulin), which bind to the cytoskeleton by F-actin and myosin II [165]. TJs have highly dynamic structures, and are capable of assembly/disassembly with different degree of sealing, depending on stimuli contexts or physiological/pathological conditions. Alteration of TJs can lead to increased paracellular transport of solutes and water, causing diarrhea, and then increased permeability to large molecules, such as luminal pathogens. As mentioned previously, alteration of TJs was already noticed in IBD patients long ago, for example the overexpression of Claudin-2 [166,167] and downregulation or redistribution of Occludin, Claudin-5, Claudin-8 [167] and Claudin-3, and Claudin-4 [166]. Even though increased TJ permeability is considered a hallmark of IBD, whether it is an initial event causing the broken barrier of the gut remains unclear. Animal studies showed that mice with increased permeability did not initiate inflammation but accelerate mucosal immune responses [55], while mice lacking predominant intestinal TJ (claudin-7) developed colitis without major barrier integrity alteration nor increased permeability of large organic solute, but rather with augmented paracellular permeability of small soluble bacteria [168]. In contrast, intestinal permeability was significantly increased even several years prior to the patient being diagnosed with CD [46], and thus it might serve as a prognostic marker for disease pathophysiology. 

Zonula occludens 1 (ZO1), a tight junction scaffold protein, was indicated to play a role in physiological cell extrusion and barrier preservation indicated by actively ZO-1 redistribution before, during, and after cell shedding [17]. ZO-1 alterations were exemplified by funnel-shaped formation of ZO-1 hugging within the dying cell; this ZO-1 patch remained after the nucleus had been completely shed into the lumen. Interestingly, the size of neighboring cells remained unchanged during the whole process, while the ZO-1 patch beneath the extruded nucleus initially appeared to have a similar size as the neighboring cells, but then reduced in size over time along with the healing process. Consistently, in TNF-induced pathological cell shedding, vital redistributions of ZO-1, occludin, and lateral junctions (Claudin-7 and Claudin-15) were also detected [18]. An experiment on human colorectal adenocarcinoma cell lines (Caco-2 cell) confirmed that cells stimulated with inflammatory stimulus (LPS) had a lower expression of ZO-1, occludin, and Cldn-1, along with induced intestinal barrier damage assessed by transepithelial electrical resistance. Beyond cell shedding, ZO-1 interacts with centrioles and contributes to the orientation of mitotic spindles to complete cellular mitosis [169]. Mice lacking ZO-1 in the IEC did not display spontaneous disease or barrier dysfunction, but showed slightly increased gut permeability, and were therefore hypersensitive to mucosal insults and depicted compromised mucosal repair after chemical injury. Interestingly, the authors showed that ZO-1 was suppressed in IBD biopsy specimens, suggesting that ZO-1 restoration in IEC could be a key step in promoting effective mucosal healing in IBD patients [169]. Accordingly, Ibrahim et al. had suggested that ZO-1 expression was negatively regulated through the NFκB pathway via PIK3R3, which indeed was found to be overexpressed in IBD patients. In DSS-treated mice, blocking PIK3R3 by TAT-N 15 showed a protective effect against inflammation development, with less disrupted TJs and reduced intestinal permeability [170]. Moreover, another recent study showed that T cell protein tyrosine phosphatase non-receptor type 2 (TCPTP) protected intestinal barriers by restricting the remodeling of ZO-1, occludin, and Cldn-2 via the upregulation of the inhibitory cysteine protease, matriptase [171]. Actually, occludin represents another TJ protein with a relevant function in cell shedding. During TNF-induced cell shedding, epithelial integrity was maintained by AJs and TJs remodeling, including the “funnel-like” structure of occludin. Interestingly, the lack of this protein in gut epithelium did not cause any essential changes in terms of intestinal structure and barrier functions [172,173], which raised a big question about the importance of occludin. In a recent study, Kou et al. had shown that mice lacking occludin in intestinal epithelium were protected from severe DSS- or TNBS-induced colitis, since the activation of both intrinsic and extrinsic apoptotic pathways were blocked. Likewise, results obtained from the analysis of IBD biopsies indicated that occludin downregulation was correlated with caspase-3 reduction, suggesting that targeting occludin could have beneficial effects on limiting epithelial damage during inflammatory conditions [174].

The gut epithelial AJs are composed of α-, β-catenin, and E-cadherin, which contribute to dynamic adhesive cell-cell contact as well as gene regulation. Among IBD susceptibility loci, studies aiming at the description of heterogeneity within the NOD2 locus show that it is associated with other regions within the chromosome 16q, such as those containing CDH1 and CDH3 genes (encoding E-cadherin and p-cadherin, respectively) [175,176]. Moreover, reduced p120-catenin and β-catenin were noticed in IBD mucosa [177], and the expression and translocation of cytoplasm/nuclear β-catenin were also different between UC and CD [178], suggesting that the alterations of AJs maybe vital for IBD development. In preclinical studies, mice lacking E-cadherin in intestinal epithelium experienced more severe inflammation upon DSS-induced acute colitis compared to control mice, despite an increased epithelial regeneration due to facilitated cell migration [179]. Moreover, mice treated with antibodies against E-cadherin reduced inflammation severity and promoted barrier function [180]. Another study also showed that Ndrg2-knockdown mice developed mild spontaneous colitis with increased colonic permeability, due to the disruption of AJs via E-cadherin expression attenuation [181]. In IBD, the depletion of Ndrg2 positively correlates with E-cadherin and negatively correlates with inflammation severity [181]. Indeed, E-cadherin has been reported to be associated with cell shedding [182,183,184]. Cells expressing a constitutively active form of CDC42 (an oncogenic mutation) enhanced extracellular metalloproteinase-mediated E-cadherin cleavage and showed extremely high rates of extrusion [183]. In addition, E-cadherin is also required for coordination of neighboring cell elongation during apoptotic cell shedding. The retraction of the apoptotic cell stimulated the elongation of surrounding cells in an E-cadherin-dependent manner [182], which requires the recruitment of coronin 1B to the junctional cortex between shed/neighbor cells to promote actin rearrangement and cell extrusion [185]. The cell elongation demonstrated in this study is contradictory to other previous studies, as mentioned above. In accordance with the relevance of EDAC to avoid tumorigenesis [20], the decline of membrane E-cadherin has also been observed in UC dysplasia, together with enriched nuclear cyclin D1 and accumulation of intracellular β-catenin [186]. In fact, the switch between N- and E-cadherin is a well-established initial phenomenon in epithelial to mesenchymal transition (EMT), and thereby, in tumor development [187]. 

Compared to numerous investigations about intestinal TJs and AJs, the function and response of desmosome in the context of IBD is less studied. Looking into the description of the contribution of desmosomes to intestinal barrier function, Petit et al. took advantage of mice lacking cellular prion protein (PrP^c^), a ubiquitous glycoprotein located in cell-cell junctions and interacting with desmosomes. PrP^c^-deficient mice showed significantly increased intestinal permeability and impaired intercellular junctions, and were therefore more sensitive to DSS-induced colitis. The distribution of PrP^c^ were also altered at cell-cell junctions in patients with IBD, highlighting the importance of desmosomes and desmosomal protein in regulating the intestinal barrier integrity and susceptibility to inflammation [188]. The adhesion at desmosomes is mediated by desmogleins (Dsg 1-4) and desmocollins (Dsc 1-3), in which only Dsg2 and Dsc2 are expressed in IECs [189]. Interestingly, the alteration of both Dsg2 and Dsc2 has been seen in CD and/or UC patients [190,191]. Mice lacking intestinal epithelial Dsg2 exhibited escalated intestinal permeability and developed a more-pronounced colitis upon DSS or *Citrobacter rodentium* challenge [190]. Furthermore, a recent study showing the close correlation between the integrity of Dsg2 and Claudin-2 expression via sequestering PI-3-kinase, confirmed the key role of Dsg2 in intestinal barrier stabilization [192]. Although initially it was considered that the loss of Dsg2 might contribute to the compromise of the epithelial barrier and therefore represents a trait in IBD, recent studies also pointed to the relevance of Dsc2. Thus, deleting Dsc2 in mouse IEC did not vastly influence intestinal mucosal architecture, but induced a delayed recovery upon DSS-induced colitis or biopsy-induced colonic wounding. Downregulation of Dsc2 was also noticed in inflamed tissue in both human UC and murine colitis [193]. In summary, the loss of desmosomes has been recognized as a trait in IBD and is believed to contribute to the compromise of the epithelial barrier.

Altogether, these studies highlight the importance of cell shedding accompanied by dynamic reorganization of junctional proteins permitting epithelial sealing, and thereby protecting mucosal contiguity and function.

## 7. Immune-Epithelial Communication

Residing in the gut lamina propria, innate and adaptive immune cells build a local immune barrier underneath the epithelial layer, as a prompt response to harmful pathogens. Under pathological conditions such as in chronic inflammation, several cytokines are released by locally activated cells. Moreover, activated immune cells express adhesion molecules, auxiliary signals, chemokine receptors, and integrins [194]. Genomic-wide associated studies (GWAS) have identified around 200 susceptibility loci associated with an increased risk for IBD; several of them encode cytokines or their downstream mediators [195,196]. Thus, these cytokine signals largely contribute to intestinal inflammation, clinical symptoms, and may also regulate IBD complications (like intestinal stenosis, fistula formation, and colitis-associated neoplasias) [197]. The pro-inflammatory milieu in the inflamed gut of IBD patients also impacts epithelial function. As a clear example of immune-epithelial communication, diverse cytokines impact on TJ disassembly. For instance, IL1B and IL6 caused redistribution and/or expression of occludin and claudin-2 in Caco2 cells, respectively [198], while T cell-derived LIGHT activated endocytosis of TJs in intestinal epithelial monolayers [199].

TNF–produced mainly by monocyte, macrophage and T cells–is a well-described mediator in diverse biological processes, including cell division, differentiation, and cell death. Actually, IBD loci associated with TNF signaling (for example: NFKB1, REL, and TNFAIP3) were also identified by GWAS [200,201]. In fact, TNF was found to promote inflammation [202] as well as colitis-associated colon carcinogenesis initiation and progression [203]. Previous work had shown that TNF-secreting cells were significantly increased in the inflamed intestinal mucosa, especially seen in CD patients [204]. Both membrane-bound and soluble TNF levels were significantly augmented in the lamina propria of IBD patients [197]. Accordingly, anti-TNF therapy is perhaps one of the most effective therapy for IBD so far, despite causing adverse side-effects. The fact that about one third of patients do not response to these agents [205] emphasizes the importance of understanding molecular mechanisms behind TNF inhibition, which might enable us to categorize IBD patients by predictive response markers and, accordingly, provide them with the most effective treatments.

Importantly, TNF is a key trigger for cell shedding, but its effects in intestinal epithelial function varies among different studies, depending on the degree of stimulation. Spontaneous and naturally occurring gaps in the gut epithelium due to cell extrusion can be detected in steady-state conditions, however only 3% of them caused leakage (Lucifer yellow penetrance) [206]. However, TNF-induced cell shedding led to increased gap formation, allowing Lucifer yellow penetrance in 20% of the cases, leading to a loss of barrier function [207]. Accordingly, another study claimed that TNF-treated mice showed alterations of barrier integrity and function due to increasing epithelial extrusion of multiple adjacent cells, followed by gap formation and microerosions, which cannot be healed by TJs redistribution of surrounding cells [44]. Paradoxically, it has been shown that even with a high dose of TNF, the barrier function surprisingly remained intact despite extensive cell shedding. This could be explained by the dynamic cytoskeleton rearrangement of intestinal epithelial cells at the shedding sites, represented by the remodeling of perijunctional actin (F-actin, ROCK, MLCK), TJ proteins (ZO-1, occludin), and apical junctional complex (ROCK, E-cadherin, Claudin-5, -7) [18]. Another reason could be the rapid healing rate of the epithelium, since in less than 20 min the epithelium quickly resumes to basal position by sealing the gap upon TNF-induced cell shedding [18]. Of interest, perfusing TNF-treated mice tissue segments with dynasore, a specific dynamin inhibitor, successfully arrested 65% of cell shedding events, suggesting the role of dynamin II-dependent membrane traffic in TJs redistribution and cell shedding [18,208]. Together, these studies demonstrate that TNF induces cell shedding with variable outcomes, probably due to uneven rates of endogenous cell shedding among murine intestines [13]. Recently, new insights about mechanisms behind TNF-induced cell shedding suggest that TNF-induced cell extrusion is controlled by IRF1 via the caspase-3-dependent pathway, and occurs in a GSDME-dependent pyroptotic manner [90] (see Section 4.3). 

Considering immune-epithelial communication, intraepithelial lymphocytes in close contact with enterocytes emerged as critical players. Recently issued studies demonstrate that Granzyme A and B produced by IEL are capable of inducing cell shedding. Using intravital microscopy approaches, the authors showed CD103-E-cadherin-mediated cell communication between IEL and IECs at sites of pathological cell shedding [209]. 

Beyond the interaction with immune cells and derived mediators, microbial-epithelial communication also affects gut epithelial cell extrusion. Thus, pathological cell shedding can be stimulated by microbial products, such as LPS-dependent induction of IEC apoptosis, in TNFR1- and NFκB2-dependent signaling. Mice injected with lipopolysaccharide (LPS) exhibited rapid IECs apoptosis and shedding after 1.5 h, followed by increased permeability and severe pathological changes [210]. In this case, excessive cell extrusion goes along with villus shortening, fluid exudation into the lumen, and diarrhea [210]. Furthermore, Günther et al. have shown that stimulation of toll-like receptors (TLR3 and TLR4) that facilitate microbial recognition could quickly activate caspase-8 and elevated cell shedding. In TLR-stimulated mice, IEC-specific caspase-8 knockdown caused RIP3-dependent necroptosis instead of apoptosis, leading to excessive cell death, tissue damage, and mortality [86]. Accordingly, blocking RIP kinases could prevent necroptosis/extrusion of infected cells and preserve mucosal integrity. Additionally, the authors pointed out that TLR3-induced necroptosis was directly regulated by the TRIF-dependent pathway (independent of TNF-α and type III interferons), and that TLR4-mediated necroptosis was highly dependent on TNF-α. Further supporting the role of the microbiota in the context of cell shedding, Hughes et al. depicted the relevance of early-life gut microbiota colonization. Strikingly, neonatal mice were protected against LPS-induced pathological cell shedding due to the different microbiota composition [211].

## 8. Conclusions, Future Perspectives

The maintenance of epithelial integrity in the gut mucosa is essential to the protection of underlying tissue compartments, due to dynamic changes of the nutritional and microbial environment. Excessive IEC shedding leads to a compromised gut barrier, which can flare up into exaggerated immune reactions associated to pathological conditions. In the past, many studies contributed to our understanding of pathological intestinal cell extrusion. Those studies focused on intrinsic or extrinsic apoptotic stimuli responses and highlighted the importance of cytoskeleton dynamics and intercellular junctions. However, the discovery of the S1P-S1P2 signaling pathway and mechanosensitive channels have shed a new light on how homeostatic cell shedding actually occurs in the body. Those studies also begin to answer two ever-pertinent questions about cell shedding: how the IEC shedding-proliferation axis is perfectly balanced despite great distance between crypt bottom and villus tip, and how a single cell is initiated to shed. Nevertheless, the function of mechanosensitive channels in mediating intestinal shedding in mammals is still missing and needs to be further explored. Basic research about mechanisms regulating cell extrusion will provide us with a deeper understanding of the pathological relevance of cell shedding. For instance, how pathogens interact with IECs and inhibit/induce cell shedding to create vulnerable penetrable sites, or how different oncogenic mutations drive apical/basal extrusion to invade underneath tissues. Another important aspect that should be considered is epithelial cell death, the escalation of which has been constantly observed as a hallmark of severe IBD, and that notably is able to restore gut barrier function in the inhibition of IEC death [212]. Each cell death form modulates cell shedding in a different way and little is known about its underlying mechanisms. Better understanding of how diverse cell death pathways and cell shedding are associated with each other is certainly required for improving IBD treatment. In conclusion, addressing and understanding cell extrusion mechanisms and its regulation, in both physiological and pathological contexts, is fundamental to broadening our knowledge of intestinal homeostasis, and may allow further development of therapeutic approaches for IBD and possibly CRCs.

## Figures and Tables

**Table 1:** Cell death pathways in the context of cell extrusion.

Cell Death	Features	Main Mediators	Inflammation	Cell Extrusion (References)
Apoptosis/Anoikis	Cell shrinkage, chromatin condensation, membrane blebbing	Caspase-3 Caspase-8	NO	Grossmann 2001 [83] Bullen 2006 [84] Marchiando 2011 [18] Andrade 2011 [85]
Necrosis	Cell rounding, cytoplasm swelling, dilated organelles, chromatin condensation	Complex crosstalk between multiple signalings	NO	Andrade 2011 [85] (no cell extrusion occurs in necrotic cells)
Necroptosis	Similar to necrosis	Necroptosome RIPK3 MLKL	YES	Günther 2015 [86] (TLR agonists induce necroptosis and cell shedding)
Pyroptosis	Chromatin condensation, DNA fragmentation, intact nuclei, membrane pore formation	Inflammasome Caspase-1 Caspase-11 Gasdermin-D	YES	Sellin 2014 [87] Broz 2014 [88] Rauch 2017 [89] Tan 2021 [90]
Ferroptosis	Mitochondrial alterations, ER stress	Iron ROS	YES	None

## Data Availability

Not applicable.
